# Cocrystal of Apixaban–Quercetin: Improving Solubility and Bioavailability of Drug Combination of Two Poorly Soluble Drugs

**DOI:** 10.3390/molecules26092677

**Published:** 2021-05-03

**Authors:** Li Zhang, Dewen Kong, Hongjuan Wang, Lingtai Jiao, Xiaoyue Zhao, Junke Song, Dezhi Yang, Haiguang Yang, Shiying Yang, Guanhua Du, Yang Lu

**Affiliations:** 1 Beijing City Key Laboratory of Polymorphic Drugs, Center of Pharmaceutical Polymorphs, Institute of Materia Medica, Chinese Academy of Medical Sciences and Peking Union Medical College, Beijing 100050, China; zhangl@imm.ac.cn (L.Z.); xieyifei@imm.ac.cn (H.W.); zhoujian@imm.ac.cn (L.J.); ydz@imm.ac.cn (D.Y.); ysy@imm.ac.cn (S.Y.); 2 Beijing City Key Laboratory of Drug Target and Screening Research, National Center for Pharmaceutical Screening, Institute of Materia Medica, Chinese Academy of Medical Sciences and Peking Union Medical College, Beijing 100050, China; kongdewen@imm.ac.cn (D.K.); zhaoxiaoyue@imm.an.cn (X.Z.); smilejunke@imm.ac.cn (J.S.); yhg@imm.ac.cn (H.Y.)

**Keywords:** drug–drug cocrystal, apixaban, quercetin, solubility, bioavailability

## Abstract

Drug combinations have been the hotspot of the pharmaceutical industry, but the promising applications are limited by the unmet solubility and low bioavailability. In this work, novel cocrystals, consisting of two antithrombotic drugs with poor solubility and low bioavailability in vivo, namely, apixaban (Apx) and quercetin (Que), were developed to discover a potential method to improve the poor solubility and internal absorption of the drug combination. Compared with Apx, the dissolution behavior of Apx–Que (1:1) and Apx–Que–2ACN (1:1:2) was enhanced significantly, while the physical mixture of the chemicals failed to exhibit the advantages. The dissolution improvements of Apx–Que–2ACN could be explained by the fact that the solid dispersion-like structure and column-shaped cage of Que accelerated the access of the solvent to the inner layer of Apx. The fracture of the hydrogen bonds of Apx, which was the joint of the adjacent Que chains, facilitated the break-up of the structures. Besides, the bioavailability of Apx–Que was increased compared with the physical mixture and Apx, and Apx–Que remained stable in high temperature and illumination conditions. Therefore, a drug–drug cocrystal of two antithrombotic agents with poor solubility was developed, which exhibited greatly improved solubility, bioavailability and superior stability, indicating a novel method to overcome the shortages of drug combination.

## 1. Introduction

Drug combination has attracted the expanding interests in pharmaceutical research in recent years. Compared with traditional drugs, drug combinations exhibit advantages in the increased patient compliance and adherence, the simplification of disease management, the reduced cost of drug products and the reduced probability of mono-therapy-induced resistant bacilli strains [1,2,3,4,5,6,7,8,9]. However, the promising applications of drug combinations are impeded by the limitations encountered in the formulation stages, such as poor solubility and stability, chemical interactions of ingredients, and decreased bioavailability.

Many approaches were conducted to improve the solubility and bioavailability of poorly soluble compounds of drug combination, for example, inclusion complex with hydroxypropyl-β-cyclodextrin (HP-β-CD), solid dispersion with highly water-soluble polymers, and micro-emulsion formulation [10,11,12,13,14,15]. Unfortunately, many of the methods are time consuming and limited by high cost. Besides, there are still more limitations including potential chemical interactions caused by introducing new materials into the formulation, and the high standard storage conditions required for the stability of the products.

Considering the practicability and atom-economic of the potential technique for industry application, drug–drug cocrystals could be an appropriate option to improve the solubility, bioavailability and pharmacological activity of target molecules, without modifying the chemical structure of drugs or introducing other materials into the formulation [16,17,18,19,20,21]. Moreover, the technique suited manufacture owing to the high efficiency, sustainability, and low cost. For example, Tramadol hydrochloride–celecoxib (a cocrystal drug for the treatment of pain) was approved by the China Food and Drug Administration (CFDA) in 2017 [22,23,24,25]. Herein, Apx–Que, a novel cocrystal of two anti-thrombotic agents, was designed based on the concept of cocrystal engineering.

**Scheme 1:**
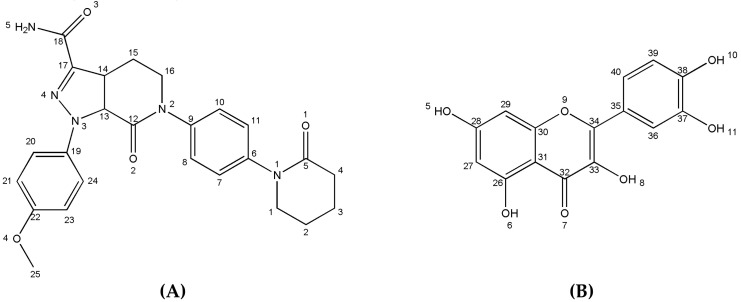
Chemical structures of apixaban (**A**) and quercetin (**B**).

Apixaban (Apx) (Scheme 1) is used to prevent venous thromboembolism (VTE) in hip or knee replacement surgery, and to decrease the risk of stroke and systemic embolism in patients with non-valvular atrial fibrillation [26,27,28,29]. Quercetin (Que) is a kind of flavonoid with an effect on reducing platelet aggregation in former studies [30,31,32,33,34,35]. Unfortunately, the potential superiority of the synergistic effect of the anticoagulant and anti-platelet drugs is restricted by the decreased solubility in physical combination as the agents are biopharmaceutical classification system (BCS) class II compounds. Therefore, cocrystal formation is adopted to improve the physicochemical properties of the drugs, and the first drug–drug cocrystal of Apx was presented, which may further improve its pharmacological activity.

The structures of apixaban–quercetin (Apx–Que) and apixaban–quercetin–2-acetonitrile (Apx–Que–2ACN) were characterized by single crystal X-ray diffraction (SXRD, Apx–Que–2ACN only), powder X-ray diffraction (PXRD), differential scanning calorimeter (DSC), and thermogravimetric analysis (TG). Compared with Apx, the solubility and bioavailability of Apx–Que were improved significantly, while the physical mixture of the chemicals failed to exhibit the advantages. The dissolution improvements of Apx–Que–2ACN could be explained by the fact that the solid dispersion-like structure and column-shaped cage of Que accelerated the access of the solvent to the inner layer of Apx, and the fracture of the hydrogen bonds of Apx, which was the joint of the adjacent Que chains, facilitated the break-up of the structures. Besides, Apx–Que exhibited excellent stability in high temperature and illumination conditions. Therefore, a drug–drug cocrystal of two antithrombotic agents with poor solubility was developed, which exhibited greatly improved solubility, bioavailability, and superior stability, indicating a novel method to overcome the shortages of drug combination.

## 2. Experimental Section

### 2.1. Materials 

The solvents in analytical grade were purchased from Beijing Chemical Works. Apx and Que were purchased from Wuhan Yuancheng Technology Inc, with qualified purification (HPLC > 99%). Carbamazepine, which served as an internal standard (IS), was purchased from J&K Scientific (Beijing, China). Mass spectrometry grades of acetonitrile and methanol were obtained from J. T. Baker (J. T. Baker, Seattle, WA, USA). Formic acid was purchased from Thermo Fisher Scientific (Beijing China). All other chemicals used were of pharmaceutical grade.

### 2.2. Experimental Animals 

Sprague Dawley (SD) rats (about 200 g, male, SPF grade) were purchased from Beijing Vital River Experimental Animal Co. Ltd. (Vital River, Beijing, China). Certificate no: SCXK (Beijing) 2016-0006. The animals were kept in an environment at 24 ± 2 °C with ad libitum access to food and water. Experimental protocols were performed in accordance with institutional guidelines for the care and use of laboratory animals at the Institute of Materia Medica, Chinese Academy of Medical Science and Peking Union Medical College and the National Institutes of Health Guide for Care and Use of Laboratory Animals (publication no. 85-23, revised 1985). 

### 2.3. Preparation of Apx–Que–2ACN

The powder sample was prepared by slurrying, and the single crystal was obtained with solution crystallization. Slurrying: a mixture of Apx (0.1 mmol, 46.0 mg) and Que (0.1 mmol, 30.2 mg) was added to 4 mL acetonitrile and ethyl acetate (1:1, v:v), and the suspension was stirred with IKA RT-10 at a rotation speed of 300 rpm for 3 h at 30 °C. After filtration, the solid was dried at 60 °C for 30 min. Solution crystallization: a mixture of Apx (0.1 mmol, 46.0 mg) and Que (0.1 mmol, 30.2 mg) was added to 50 mL acetonitrile and ethyl acetate (1:1, v:v). The suspension was heated to 60 °C, remaining for 15 min, and cooled down to room temperature. After filtration, the solution was crystallized at ambient temperature. A yellow plate-shaped crystal suitable for SXRD study was harvested after 30 days.

### 2.4. Preparation of Apx–Que 

The powder sample was obtained from Apx–Que–2ACN, which was dried at 130 °C, 0.02 MPa with DZF-6020 (Shanghai Yiheng Technology Co., Ltd, Shanghai, China.) for 1 h and the solvent was removed completely. 

### 2.5. SXRD 

SXRD study of Apx–Que–2ACN was conducted at 293 K on a Rigaku MicroMax-002+ diffractometer (Rigaku, Tokyo, Japan) using Cu*Kα* radiation (λ = 1.54178 Å). The structure was solved by direct method and refined with full-matrix least-squares technique using OLEX2. Non-hydrogen atoms were refined with anisotropic displacement parameters. Hydrogen atoms were placed in calculated positions and refined with the riding model, and the protons on OH and NH were located from difference Fourier maps.

### 2.6. PXRD 

Patterns of PXRD were obtained on a Rigaku D/max-2550 diffractometer (Rigaku, Tokyo, Japan) using Cu*Kα* radiation, and the voltage and current of the generator were set to 40 kV and 150 mA. Patterns were collected in the 2 θ range of 3–40° with a scan rate of 8° min^−1^ at ambient temperature, and the data were imaged, integrated and analyzed with Jade 6.

### 2.7. DSC

DSC was performed on Mettler Toledo DSC/DSC1 (Mettler Toledo, Zurich, Switzerland) equipment. The samples weighing 3–5 mg in a standard aluminum pan (40 μL) were heated from 30 °C to 300 °C at the rate of 10 °C min^−1^ under a nitrogen gas flow of 50 mL/min. 

### 2.8. TGA 

TGA analysis was performed on Mettler Toledo DSC/TGA1 (Mettler Toledo, Zurich, Switzerland) equipment with a flow of 50 mL/min of nitrogen as dry air. The samples were heated in the range of 30 °C to 500 °C at the rate of 10 °C min^−1^. The STAR software package (Mettler Toledo) was employed to record and analyze the TGA and DSC curves.

### 2.9. Stability Test 

Approximately 50 mg Que–Apx in open containers was placed in the conditions of high temperature (60 ± 5 °C), high humidity (25 ± 5 °C, 90 ± 5%), and illumination (4500 ± 500 lx) for 10 days to investigate the stability via PXRD.

### 2.10. High Performance Liquid Chromatography (HPLC) Conditions

The concentration of samples in dissolution studies and intrinsic dissolution measurements were measured on an Agilent 1200 (Agilent, California, USA) with a diode array detector. The mobile phase was acetonitrile: 0.4% phosphoric acid = 30:70 (v:v), and a Kromasil 100-5-C18 column (Kromasil, Sweden) (250 mm × 4.6 mm, 5 um) was used as an analytic column; chromatograms were monitored at 254 nm and isocratic elution was used for all processes. The retention time of Apx and Que was 17.13 and 12.57 min, respectively.

### 2.11. Powder Dissolution Measurements

Powder dissolution measurements and intrinsic dissolution measurements were performed on an RC12AD (Tianjin TIANDA TIANFA pharmaceutical testing instrument manufacturer, Tianjin, China) with an automatic sampling system RZQ-12D (Tianjin TIANDA TIANFA pharmaceutical testing instrument manufacturer, Tianjin, China), where an equal volume of the blank dissolution media would be injected automatically into the dissolution vessels after sampling. The powder samples of Apx, physical mixture, Apx–Que, and Apx–Que–2ACN were milled at a rotation speed of about 60 rpm for 5 min with a agate mortar and pestle (Shanghai Lichen Bangxi Instrument Technology Co., Ltd., Shanghai, China), and sieved through 100-mesh sieves (Shaoxing Highway Instrument Equipment Co., Ltd.) in 2 min to minimize the size effect on results. Accurately weighed Apx (30.0 mg), physical mixture (49.7 mg), Apx–Que (49.7 mg), and Apx–Que–2ACN (55.2 mg), equivalent to 30.0 mg Apx, were added to dissolution vessels containing 450 mL media, namely, pure water, 0.1 M HCl, acetate buffer saline (ABS) of pH 4.5, and phosphate buffer saline (PBS) of pH 6.8. The suspensions were stirred at a rotation speed of 100 rpm at 37 °C, and sampled at 0, 5, 15, 30, 60, 90, 120, 180, 240, 360, and 480 min. The samplings were filtered through 0.22 μm PTFE filters, and the concentration of Apx was determined by HPLC. The remaining solids were filtrated and detected by PXRD, and the pH of the media after dissolution was also measured by pH Meter (Mettler Toledo, S210, Mettler Toledo, Zurich, Switzerland). The powder dissolution experiments were carried out in triplicate to evaluate the standard deviations.

### 2.12. Intrinsic Dissolution Measurements 

Accurately weighed Apx (60.0 mg), physical mixture (99.4 mg), Apx–Que (99.4 mg), and Apx–Que–2ACN (110.4 mg), equivalent to 60.0 mg Apx, were compressed with a hydraulic press FODT-101Y (Shanghai Fukesi Analytical Instrument Co., Ltd., Shanghai, China) at 51.0 MPa (100 kg on a 0.196 cm^2^ disk) for 10 min to obtain a 5 mm diameter disk. One surface of the disk was sealed and the other one was exposed to the medium. The samples were added to dissolution vessels containing 450 mL pure water. The suspensions were stirred at a rotation speed of 100 rpm at 37 °C, and sampled at 0, 5, 10, 15, 20, 25, 30, 40, 50, and 60 min. The samplings were filtered through 0.22 μm PTFE filters, and the concentration of Apx was determined by HPLC. The IDR experiments were carried out in triplicate to evaluate the standard deviations.

### 2.13. Pharmacokinetic Study 

#### 2.13.1. Chromatographic Conditions

A high-performance liquid chromatography (Agilent 1260, Agilent, CA, USA) system equipped with an Agilent SB-C18 column (50 × 2.1 mm, 2.7 μm) was used for analysis. The mobile phase consisted of acetonitrile: water containing 0.1% formic acid = 50:50 (*v*/*v*) with a flow rate of 0.3 mL/min. The column temperature was set at 35 °C, and the elution time was 3 min. Within the ESI^+^ mode, protonated forms [M + H]^+^ at *m/z* 460.0 and 237.0 were chosen as precursor ions for apixaban and IS, with a fragment voltage of 160 V and 105 V, respectively. 

#### 2.13.2. Method Validation

The quantitative methods were validated with reference to the FDA guidelines on specificity, linearity, precision, accuracy, recovery, and stability compliance.

To ensure that endogenous and other substances in the sample would not interfere with the analysis of Apx and IS, the selectivity of the method was evaluated by comparing the blank rat plasma and corresponding spiked plasma chromatograms.

A calibration chart (n = 8) was constructed by plotting the peak area ratio of Apx to IS (y-axis) as a function of Apx (x-axis) concentration. Calculate the linear relationship of each standard curve by a weighted (1/x^2^) least squares linear regression and keep the concentration of the sample within the linear range.

The intra-day and inter-day accuracy and precision were determined by measuring three concentration levels (0.025, 0.6, 1.25 mg·L^−1^) of Apx in a single day and three consecutive days repeatedly. Accuracy and precision were defined as relative error (RE%) and percentage relative (RSD%), respectively, and the RE% is expected to be within 15%.

Comparing the peak area of the analyte in extracted samples with the corresponding untreated standard solution, the absolute recovery was measured. Finally, the stability of quality control samples (QCs) in biological samples was assessed in different storage conditions, including at 25 °C for 24 h and three freeze–thaw at −80 °C cycles.

#### 2.13.3. Pharmacokinetics 

Apx, physical mixture, and Apx–Que were orally administered at the dosage of 60 mg·kg^−1^ (the equivalent amount of free apixaban for cocrystal) in rats. Blood samples were collected from the ocular vein into heparinized tubes at the following time intervals: 0.25, 0.5, 1, 2, 3, 4, 6, 8, 10, 12, and 24 h after administration. The samples were centrifuged at 6000 rpm (10 min, 4 °C), and the supernatants of samples were stored at −80 °C until analysis. Plasma (100 μL) was mixed with 10 μL solution of carbamazepine (1 mg·L^−1^, used as the internal standard, IS) and 290 μL of acetonitrile. After centrifugation (10 min, 1340 rpm), 10 μL of supernatant was injected into the LC-MS for analysis. The drug concentration data of Apx in the plasma were completed by DAS 2.0 software and were expressed as the mean ± standard deviation (mean ± SD).

## 3. Results and Discussion

### 3.1. Crystal Structure Analysis 

**Figure 1:**
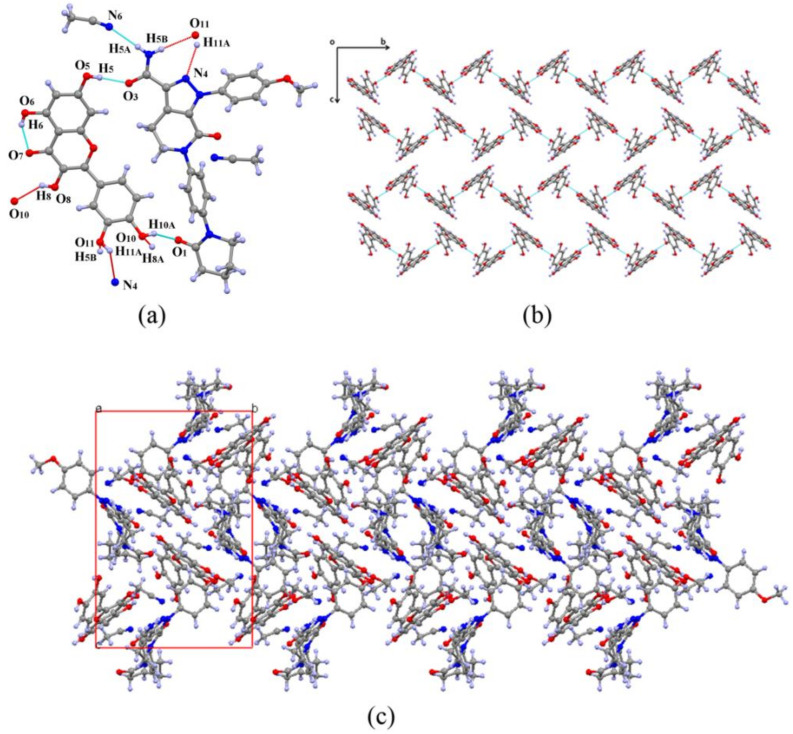
Crystal structure of Apx–Que–2ACN. (**a**) Asymmetric unit and hydrogen bonds of Apx–Que–2ACN. (**b**) Chain structure of Que. (**c**) Crystal packing network.

The structure was solved by the direct method and refined with the full-matrix least-squares technique using OLEX2. Non-hydrogen atoms were refined with anisotropic displacement parameters. Hydrogen atoms were placed in calculated positions and refined with the riding model, and the protons on OH and NH were located from difference Fourier maps. Apx–Que–2ACN was crystallized in the monoclinic space group P2_1_/n with one Apx, one Que, and two acetonitrile molecules in its asymmetric unit (Figure 1a). Crystallographic data are summarized in Table 1. The adjacent Que molecules were joined through O_8_-H_8A_···O_10_ (2.786 Å), generating a 1-D chain structure (Figure 1b) along the b axis, and an intramolecular hydrogen bond O_6_-H_6_···O_7_ (2.648 Å) was formed in Que (Table 2).

**Figure 2:**
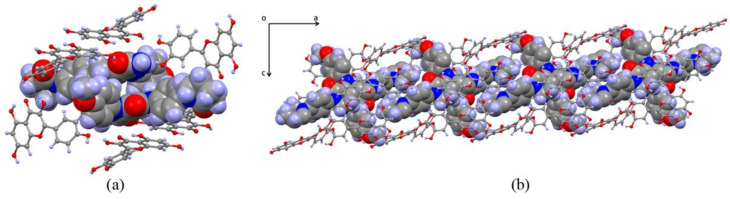
Column-shaped cage in Apx–Que–2ACN. (**a**) Que cage. (**b**) Channel along a axis.

Apx was connected to two Que molecules via hydrogen bonds and filled in the voids among Que chains. Apx and the first Que were connected with hydrogen bonds O_11_-H_11A_···N_4_ (2.878 Å) and N_5_-H_5B_···O_11_ (3.180 Å) of motif $R_{2}^{2}\left(7\right)$, and linked the second Que with O_5_-H_5_···O_3_ (2.698 Å) and O_10_-H_10A_···O_1_ (2.615 Å). The hydrogen bonds were formed owing to the similar size of the two molecules, so that the distance between the carbonyl groups (hydrogen donors) of Apx and hydroxyl groups (hydrogen receptors) of Que was suitable to form a pair of hydrogen bonds. An acetonitrile molecule was related to Apx by N_5_-H_5A_···N_6_ (3.053 Å). Interestingly, no hydrogen bond was found among Apx molecules, and the structure was similar to solid dispersion so that the chains of Que could be regarded as a carrier, which Apx was attached to (Figure 1c). In addition, two Apx were captured by six Que molecules with a column-shaped cage, which formed a channel along a axis owing to the layer structure (Figure 2).

### 3.2. PXRD Analysis 

**Figure 3:**
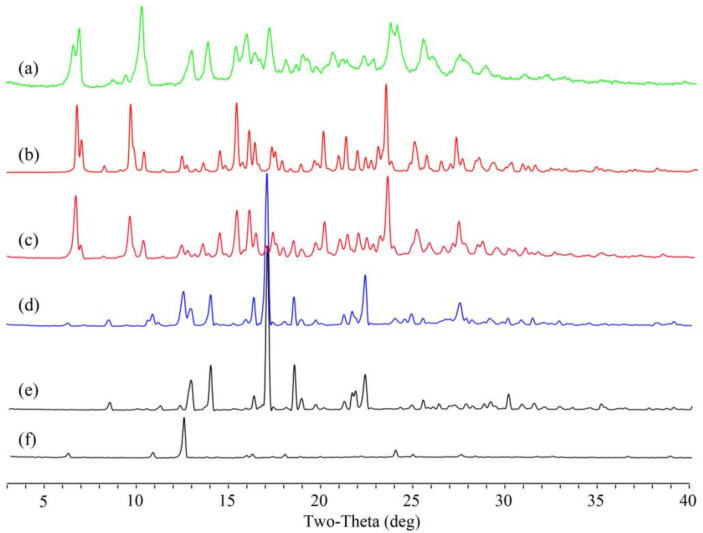
PXRD pattern of (**a**) experimental Apx–Que, (**b**) simulated Apx–Que–2ACN, (**c**) experimental Apx–Que–2ACN, (**d**) physical mixture, (**e**) Apx, and (**f**) Que.

Powder samples of Apx–Que–2ACN and Apx–Que were studied by PXRD to confirm the formation of the cocrystals. The patterns of Apx–Que–2ACN and Apx–Que are shown in Figure 3.

**Figure 4:**
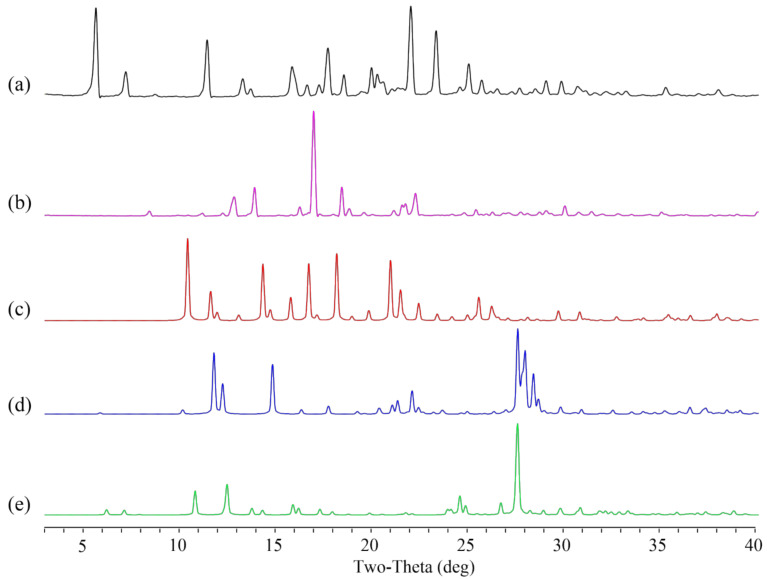
Original and other reported crystal forms of Apx and Que. (**a**) Form H2 and (**b**) N-1 of Apx, (**c**) Form I of Que, (**d**) Que hydrate, and(**e**) Que dihydrate.

The PXRD results showed that there were diffraction peaks at 2θ values of 8.54, 11.32, 12.98, 14.04, 17.08, 18.52, 21.36, and 22.38° in Apx, and 6.36, 10.90, 12.59, 15.06, 15.32, 18.04, and 24.12° in Que. Diffraction peaks at 2θ values of 6.36, 8.54, 10.90, 12.59, 14.04, 17.08, 18.52, 22.38, and 27.30° could be found in the physical mixture of Apx and Que. New diffraction peaks at 2θ values of 6.82, 6.92, 10.30, 12.98, 13.84, 15.32, 15.92, 16.36, 20.50, 22.72, 23.64, 25.48, 28.10, and 29.42° were shown in Apx–Que, while the characteristic peaks of Apx and Que disappeared. The experimental profile of Apx–Que–2ACN was in good agreement with the simulated ones, indicating the high purity of the powder sample. Completely new diffraction peaks at 2θ values of 6.76, 7.00, 9.64, 10.34, 14.42, 15.32, 16.30, 16.72, 23.32, and 27.08° were present in Apx–Que–2ACN. The experimental patterns of Apx–Que and Apx–Que–2ACN differed from the original and other reported crystal forms of single components (Figure 4).

### 3.3. Thermal Analysis

**Figure 5:**
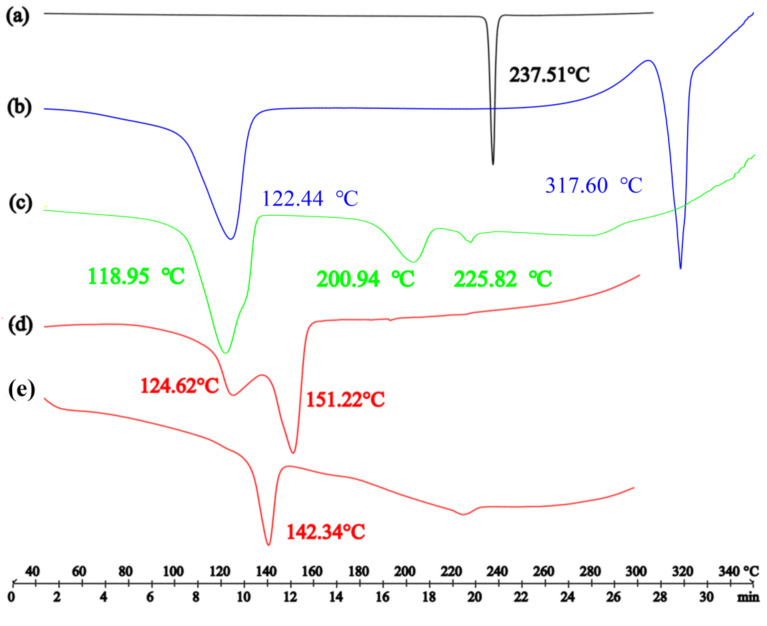
The DSC patterns of (**a**) Apx, (**b**) Que, (**c**) physical mixture of Apx and Que, (**d**) Apx–Que–2ACN, and (**e**) Apx–Que.

**Figure 6:**
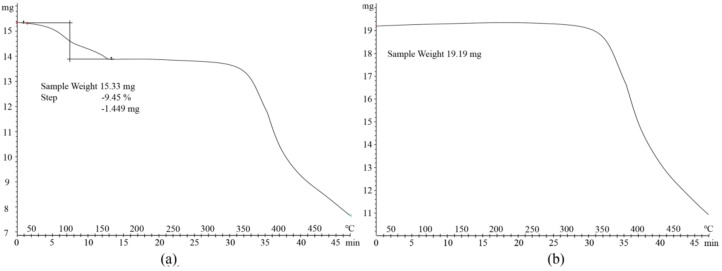
The TGA patterns of (**a**) Apx–Que–2ACN and (**b**) Apx–Que.

The DSC results (Figure 5) showed that the endothermic peak of Apx was at 237.51 °C [36], and endothermic peaks of crystal water and the melting point of Que were at about 122.44 and 317.60 °C [37], respectively. The endothermic peaks of the physical mixture were at about 118.95, 200.94 and 225.82 °C. There were studies about the salt solvate and cocrystal solvate that the endothermic peak of acetonitrile verified from 83 °C to 148 °C [38,39]. The endothermic peaks of acetonitrile and the melting of Apx–Que–2ACN, the PXRD pattern of which was in good agreement with the simulated ones, were at 142.34 and 151.05 °C, respectively, where part of the two endothermic peaks overlapped. DSC measurements confirmed the cocrystal formation of Apx–Que through a sharp endothermic peak at 142.34 °C, which indicated that the physicochemical properties of Apx–Que differed from the original chemicals and the physical mixture. TG analysis [40,41,42,43,44,45] was conducted to ensure the content of ACN in Apx–Que–2ACN was consistent with the SCXRD result that two acetonitrile molecules were included in its asymmetric unit. The weight loss in the TGA pattern of Apx–Que–2ACN was 9.45%, equivalent to 1.94 acetonitrile molecules, which was in good agreement with the SCXRD result. Apx–Que was prepared by heating Apx–Que–2ACN at 130 °c, 0.02 MPa for 1 h, and the TG experiment (Figure 6) was performed to ensure acetonitrile was removed completely. No weight loss observed in the TG analysis of Apx–Que indicated Apx–Que–2ACN was transformed into Apx–Que thoroughly.

### 3.4. Stability Analysis 

**Figure 7:**
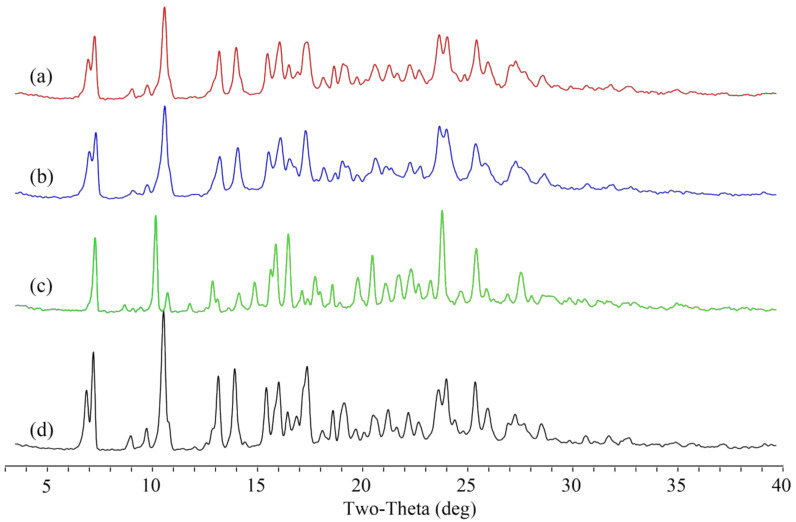
The PXRD patterns of stability studies of Apx–Que. (**a**) High temperature, (**b**) illumination, (**c**) high humidity, and (**d**) original Apx–Que.

The stability of Apx–Que in extreme conditions was studied and the results are shown in Figure 7. The powder samples in high temperature and illumination conditions remained stable after 10 days, but not stable in high humidity conditions, which illustrated that the products needed dry storage at room temperature.

### 3.5. Powder Dissolution Measurements and IDR Studies 

**Figure 8:**
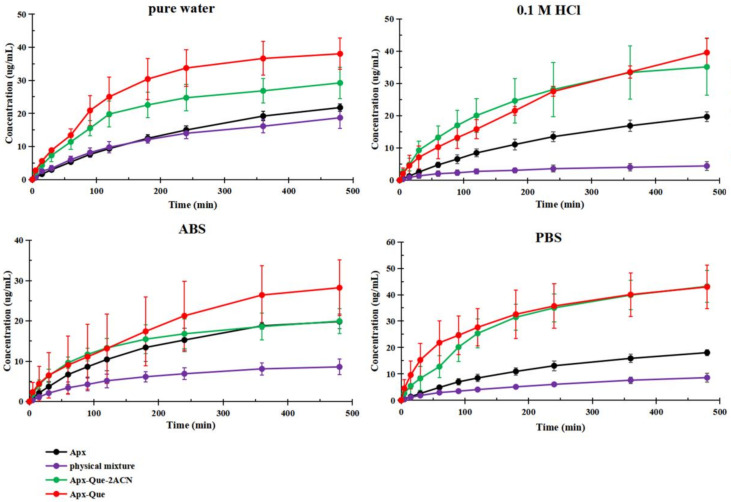
Powder dissolution profiles of Apx, physical mixture, Apx–Que–2ACN, and Apx–Que.

Improving the dissolution behavior of drugs is important to expand the applications of drug combination, as improved apparent solubility can result in enhanced bioavailability. The solubility of Apx–Que–2ACN and Apx–Que was significantly improved in dissolution media compared with pure Apx, while the mixture decreased vastly. The measurements are presented in Figure 8. The maximum concentration of the physical mixture decreased 14% in pure water, 78% in 0.1 M HCl, 57% in ABS, and 52% in PBS. Meanwhile, the maximum concentration of Apx–Que–2ACN was approximately 1.3 times higher than Apx in pure water, 1.8 times in 0.1 M HCl, and 2.4 times in PBS, and Apx–Que was approximately 1.7 times higher than Apx in pure water, 2.0 times in 0.1 M HCl, and 2.4 times in PBS. The enhanced dissolution behavior of the cocrystals lasted for more than 8 h, which could lead to improved bioavailability.

**Figure 9:**
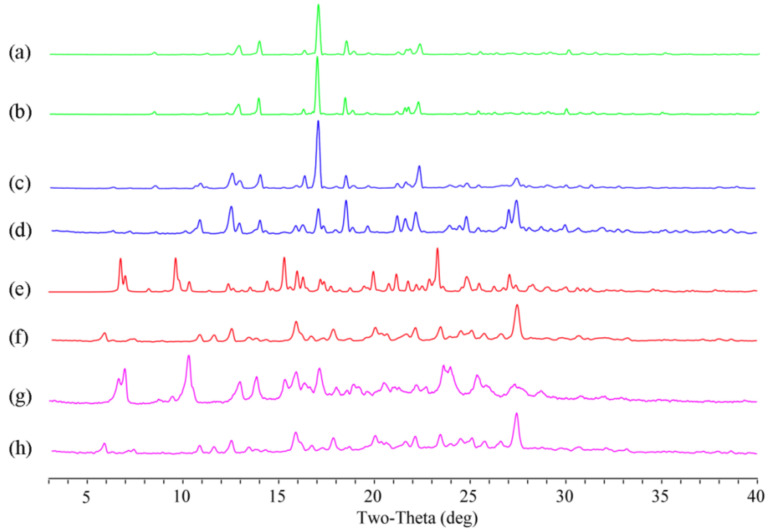
PXRD patterns of samples before and after dissolution. Green lines for Apx, blue lines for physical mixture, red lines for Apx–Que–2ACN, and pink lines for Apx–Que.

The formation of samples after dissolution studies was examined through PXRD, which showed that Apx and the physical mixture were stable in dissolution media during the solubility study, but Apx–Que and Apx–Que–2ACN were transformed into the mixture of Apx (Form H2) and Que in the process (Figure 9). The pH of the media after dissolution studies is summarized in Table 3.

**Figure 10:**
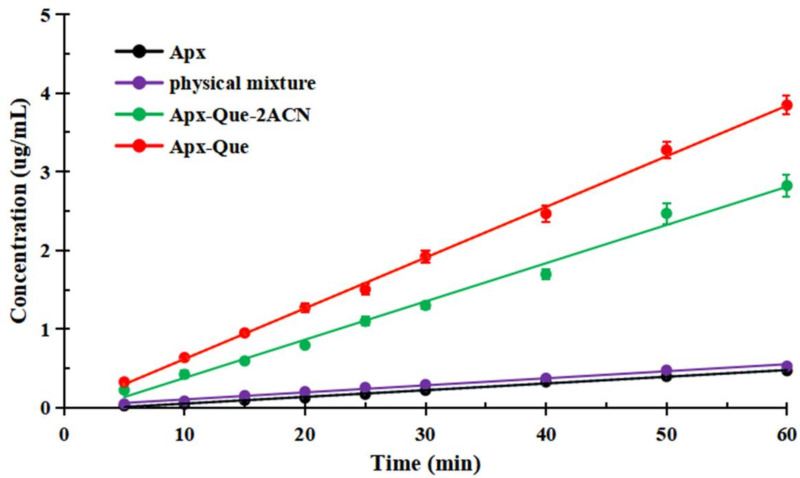
IDR profiles of Apx, physical mixture, Apx–Que–2ACN, and Apx–Que.

It is known that IDR is with a better correlation in in vivo drug dissolution dynamics than powder solubility. In order to obtain quantitative information of the dissolution rates, IDR experiments for the samples were performed and the curves of cocrystals were substantially different from Apx (Figure 10). The result showed that the dissolution rate of Apx in physical mixture did not exhibit advantage. However, the release of Apx in Apx–Que–2ACN and Apx–Que was about 6 and 8 times higher than pure Apx, respectively, which indicated the probability of cocrystals to improve the absorption and bioavailability.

The results indicated that the solubility of Apx was not improved in the formation of physical mixture owing to the poor solubility of Que in water that insoluble Que attached to Apx and decreased the contact area of water and Apx, which resulted in a decreased dissolution rate. However, the dissolution behavior of the cocrystals was improved significantly in the dissolution studies, which could be explained by the fact that the permutations in cocrystals differed from the original components. In Apx–Que–2ACN, Apx was captured by Que molecules and attached to the chains with hydrogen bonds, which formed a solid-dispersion structure and changed the hydrogen bonds among Apx and Que molecules. Moreover, the column-shaped cage generated a channel which helped the access of the media to inner layer Apx molecules. Besides, Apx was the joint of the adjacent Que chains and the fracture of the hydrogen bonds during the dissolution accelerated the breakup of the structure. When an Apx molecule was dissolved, the neighboring Apx would be exposed to the media fully, which facilitated the dissolution. 

It is an interesting phenomenon in the dissolution studies that the physical mixture was not significantly different with the pure drug in water, but the two profiles are completely different in acetate buffer, 0.1 M hydrochloric acid, and phosphate buffer. There were studies showing that the solubility of the drug could be influenced by the different buffers and pH of the media [46,47]. Balvant Yadav [48] found that the solubility of isoniazid differed from the physical mixture and the cocrystal of isoniazid and quercetin in water, acetate buffer, 0.1 M hydrochloric acid, and phosphate buffer. Shuzhen Ren [49] found that the pH of the media influenced the solubility of pure components and cocrystals. In our studies, the pH of the water after dissolution studies (Table 3) of Apx and the physical mixture was 6.5 and 6.1, respectively, which indicated that the media environment of the physical mixture was different compared with that of pure Apx. Meanwhile, the pH of the other three buffers after dissolution studies was not changed compared with the original ones, which may lead to the different result in pure water compared with the other three buffers. Moreover, the irons in the four media were different, and the properties may influence the solubility of the pure component, physical mixture and cocrystals that the dissolution results of samples in four media were significantly different. 

### 3.6. Pharmacokinetic Study 

#### 3.6.1. Method Validation

**Figure 11:**
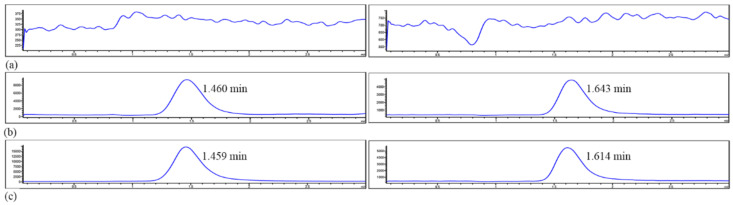
LC-MS chromatograms of Apx (left) and IS (right) obtained by deproteinization of (**a**) blank plasma, (**b**) blank plasma spiked with Apx and IS, (**c**) plasma sample after oral administration of Apx spiked with IS.

The separation time of Apx and IS was 1.4 and 1.6 min, respectively. The peak shape was presented well with acceptable baseline noise and selectivity. No interference of endogenous or other substances was detected (Figure 11).

The calibration curve displayed good linearity (R^2^ = 0.9995) within the concentration range of 0.01–1.5 mg·L^−1^ for Apx, and the regression equation of the plot was y = 0.004 x − 0.02. The intra-day and inter-day accuracy (RE %) ranged from −0.64 to 4.49%, and the precision (RSD %) was lower than 5.94%.

The recovery is used to evaluate the ability to extract analytes from biological samples. In this study, the recoveries of Apx at three concentrations were (108.48 ± 0.76) %, (105.32 ± 38.31) % and (102.20 ± 49.98) %, with an RSD of 2.82%, 6.06% and 3.91%, respectively. Under different storage conditions, Apx has an RE range of 12.32% to 4.93% and an RSD of 0.18% to 4.65%, indicating that the developed method was reliable and reusable for quantitative analysis.

#### 3.6.2. Pharmacokinetic Analysis

**Figure 12:**
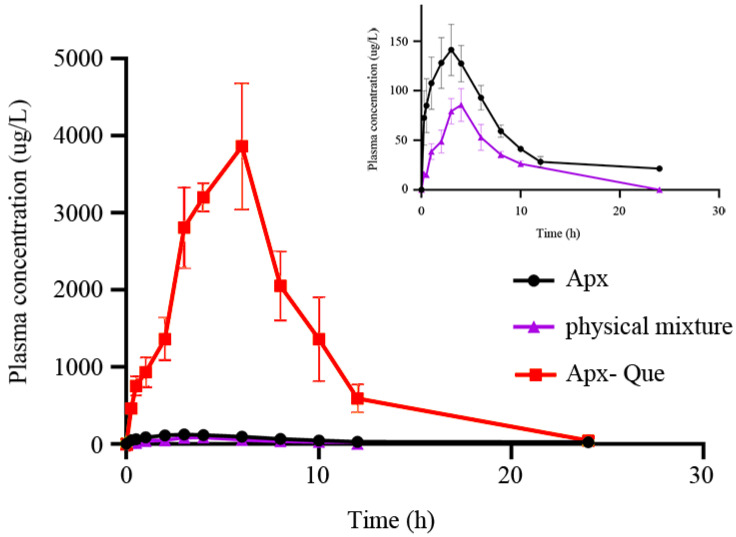
Mean plasma concentration–time curves of oral Apx, physical mixture, and Apx–Que in SD rats (x ± s, n = 5).

Pharmacokinetics in male rats was performed to evaluate the bioavailability improvement of the cocrystal. The plasma concentration after oral administration was measured. The plasma concentration–time profiles of Apx, the physical mixture, and Apx–Que are shown in Figure 12, and the mean pharmacokinetic parameters are summarized in Table 4. The plasma concentration of Apx–Que was improved significantly compared with Apx and the physical mixture. The C_max_ and AUC_0-t_ of Apx–Que increased 28.89 and 31.24 times compared with Apx, while those of the physical mixture decreased to 0.68 and 0.42 times. The result could be explained by the different dissolution behavior of the materials and that the enhanced solubility of Apx–Que accelerated the absorption and the physical mixture failed to improve the bioavailability because of its decreased dissolution, indicating that different formations influenced the pharmacokinetics of the chemicals and cocrystals could be adopted to improve the oral bioavailability of Apx. Moreover, the T_max_ of Apx–Que delayed, and the enhanced plasma concentration of Apx–Que lasted over 24 h. Notably, the clearance of Apx–Que decreased significantly compared to Apx and the physical mixture, which may also be one of the important reasons for its high Apx concentration.

## 4. Conclusions

Herein, we developed Apx–Que, a drug-drug cocrystal of antithrombotic agents with poor solubility, providing an alternative method for the innovative modification of drug combinations, with the probability to improve the safety and efficacy compared with pure Apx. Compared with Apx, the dissolution behavior of Apx–Que and Apx–Que–2ACN was enhanced significantly and the bioavailability of Apx–Que was increased compared with Apx, which illustrated that it was meaningful to study drug–drug cocrystals for pharmaceutical development. The improvements of Apx–Que–2ACN could be explained by the fact that the solid dispersion-like structure and column-shaped cage of Que accelerated the access of the solvent to the inner layer of Apx, and the fracture of hydrogen bonds of Apx, which was the joint of the adjacent Que chains, facilitated the break-up of the structure. Besides, Apx–Que exhibited excellent stability in high temperature and illumination conditions. Considering the practicability and atom-economic of the potential technique for industry application, the method suits manufacture owing to the high efficiency, sustainability, stability, and low cost, and it can be concluded that the drug–drug cocrystal is a promising approach to expand the applications of drug combinations without modifying the chemical structures of drugs or introducing other materials in the formulation.

## Figures and Tables

**Table 1:** Crystallographic data of Apx–Que–2ACN.

Compound	Apx–Que–2ACN
Empirical formula	C_44_H_41_N_7_O_11_
T (K)	293
Crystal system	monoclinic
Space group	P2_1_/n
a (Å)	17.115
b (Å)	12.888
c (Å)	19.500
α (deg)	90.00
β (deg)	92.06
γ (deg)	90.00
Volume (Å^3^)	4298
Z	4
Calculated density (g/cm^3^)	1.304
Absorption coeff. (mm^−1^)	0.794
F (000)	1768
Crystal size (mm)	0.2×0.2×0.2
Rint	0.0710
R_1_ [I>2σ(I)]	0.0712
wR_2_ (all data)	0.2170
GOF	1.087

**Table 2:** Hydrogen bonds of Apx–Que–2ACN.

D-H···A	*d* (D···A) (Å)	∠ (DHA) (deg)	Symmetry Code
N_5_-H_5A_···N_6_	3.053	172.33	[-x+1, -y, -z+1]
N_5_-H_5B_···O_11_	3.180	165.31	[x+1/2, -y+1/2, z+1/2]
O_5_-H_5_···O_3_	2.698	173.81	[x, y+1, z]
O_6_-H_6_···O_7_	2.648	148.68	
O_8_-H_8A_···O_10_	2.786	141.29	[-x+1/2, y-1/2, -z+1/2]
O_11_-H_11A_···N_4_	2.878	135.87	[x-1/2, -y+1/2, z-1/2]
O_10_-H_10A_···O_1_	2.615	160.38	[x, y+1, z ]

**Table 3:** pH of the media after dissolution.

Samples	Original Sample	Apx	Physical Mixture	Apx–Que–2ACN	Apx–Que
Pure water	7	6.5	6.1	6.2	6.0
0.1 M HCl	1.1	1.1	1.1	1.1	1.1
ABS	4.5	4.5	4.5	4.5	4.5
PBS	6.8	6.8	6.8	6.8	6.8

**Table 4:** Pharmacokinetic parameters after oral administration of Apx, physical mixture, and Apx–Que (60 mg/kg) in rats (x ± s, n = 5).

Parameter	Apx	Physical Mixture	Apx–Que
AUC_0-t_ (μg·L^−1^·h)	1164.92 ± 314.64	485.77 ± 146.96	28,994.69 ± 7555.7
AUC_0-__∞_ (μg·L^−1^·h)	1363.98 ± 316.70	677.07 ± 134.62	29,173.15 ± 7646.13
MRT_0-t_ (h)	6.26 ± 1.8	4.69 ± 0.64	6.41 ± 1.22
MRT_0-__∞_ (h)	10.33 ± 5.89	7.36 ± 2.03	6.54 ± 1.234
t_1/2_ (h)	6.89 ± 4.51	3.92 ± 2.11	3.02 ± 0.46
T_max_ (h)	2.80 ± 1.30	4.17 ± 1.94	5.40 ± 1.34
CL_z_/F (L·h^−1^·kg^−1^)	46.24 ± 12.20	91.22 ± 15.84	2.19 ± 10.66
V_z_/F (L·kg^−1^)	0.428 ± 0.234	0.494 ± 0.239	0.009 ± 0.004
C_max_ (μg·L^−1^)	141.18 ± 47.78	95.67 ± 39.14	4551.72 ± 932.91

## Data Availability

Date of the compounds are available from the authors.

## Sample Availability

Samples of the compounds are available from the authors.
